# Supplementary figures and images for: Expression characteristics and interaction networks of microRNAs in spleen tissues of grass carp (Ctenopharyngodon idella)
Source: PLoS One. 2022 Mar 28;17(3):e0266189. doi: 10.1371/journal.pone.0266189 (PMC8959171; doi:10.1371/journal.pone.0266189)

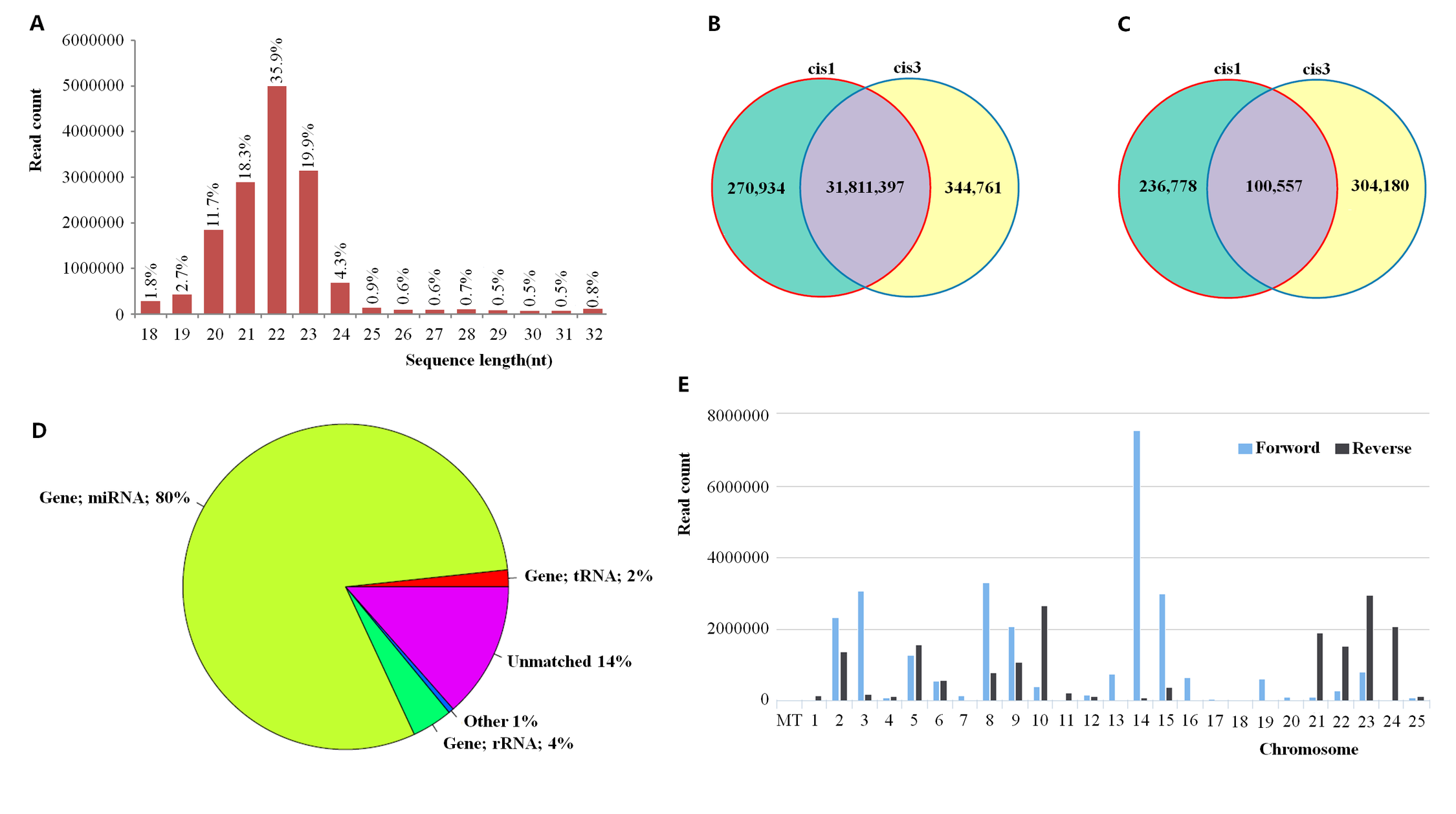

Supplement: S1 Fig — (A) Length distribution of clean reads. (B) Venn diagram of total sRNA between the cis1 and cis3. (C) Venn diagram of unique sRNA between the cis1 and cis3. (D) Annotation of unique sRNA in the RFam 11.0 database. (E) Number and distribution of clean reads mapped to the reference genome sequence. (TIF) [file pone.0266189.s001.tif]

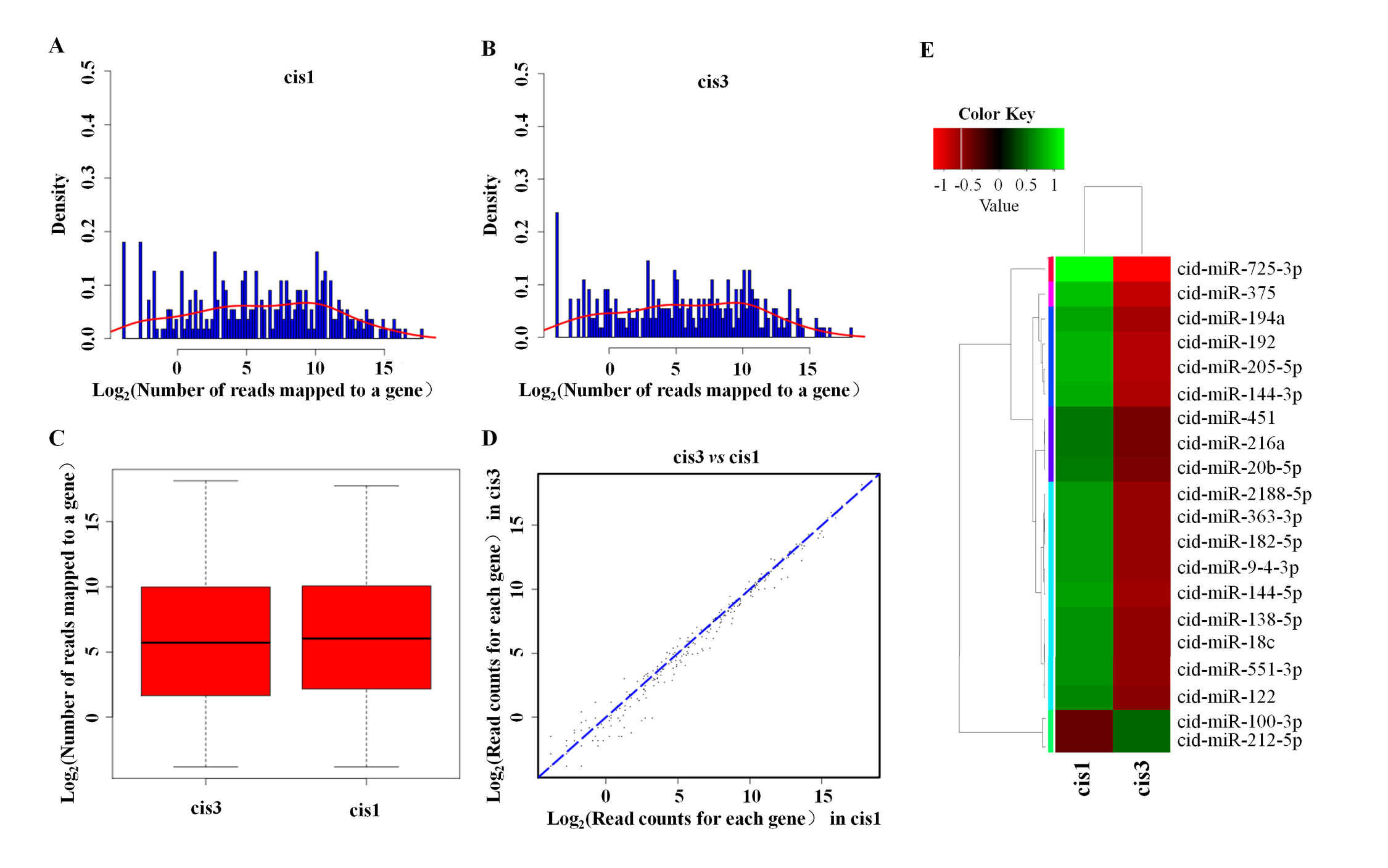

Supplement: S2 Fig — (A) Histogram of miRNA expression distribution in cis1. (B) Histogram of miRNA expression distribution in cis3. (C) Boxplot of miRNA expression distribution in cis1 and cis3. (D) Scatterplot comparing the number of miRNA reads for cis1 and cis3. (E) Heatmap of the differentially expressed miRNAs between cis1 and cis3. (TIF) [file pone.0266189.s002.tif]
