# Supplementary material for: Expression characteristics and interaction networks of microRNAs in spleen tissues of grass carp (Ctenopharyngodon idella)
Source: PLoS One. 2022 Mar 28;17(3):e0266189. doi: 10.1371/journal.pone.0266189 (PMC8959171; doi:10.1371/journal.pone.0266189)
Supplement: S1 Table — (DOCX) [file pone.0266189.s003.docx]

**S1 Table. The conserved miRNAs expressed in the spleen of grass carp.**

| **miRNA_name** | **TPM value in cis1** | **TPM value in cis3** | **miRNA_name** | **TPM value in cis1** | **TPM value in cis3** |
| --- | --- | --- | --- | --- | --- |
| cid-miR-451^＃^ | 36096.07 | 16792.92 | cid-miR-17a-2-3p^＃^ | 4.84 | 2.43 |
| cid-let-7e^＃^ | 17646.64 | 19763.12 | cid-miR-92a-2-5p^＃^ | 4.54 | 2.64 |
| cid-miR-126b-5p^＃^ | 14291.10 | 10858.03 | cid-miR-2187-3p^＃^ | 4.47 | 6.94 |
| cid-miR-731^＃^ | 9121.19 | 9509.43 | cid-miR-2192^＃^ | 3.87 | 3.68 |
| cid-miR-126a-3p^＃^ | 6660.78 | 5821.52 | cid-miR-200a-5p^＃^ | 3.35 | 2.85 |
| cid-miR-16b^＃^ | 4054.10 | 3635.36 | cid-miR-301b-5p^＃^ | 3.13 | 3.47 |
| cid-miR-144-3p^＃^ | 3317.98 | 1091.20 | cid-miR-135b-3p^＃^ | 2.68 | 3.96 |
| cid-miR-223^＃^ | 3313.21 | 4900.77 | cid-miR-7b^＃^ | 2.38 | 8.19 |
| cid-miR-181a-3p^＃^ | 2860.96 | 5182.18 | cid-miR-19c-5p^＃^ | 2.38 | 0.56 |
| cid-miR-142a-3p^＃^ | 2179.83 | 1735.05 | cid-miR-499-3p^＃^ | 1.94 | 0.83 |
| cid-miR-1388-3p^＃^ | 1760.13 | 2338.35 | cid-miR-735-5p^＃^ | 1.86 | 0.35 |
| cid-miR-199-3-3p^＃^ | 1506.14 | 1538.14 | cid-miR-10a-3p^＃^ | 1.71 | 1.87 |
| cid-miR-145-3p^＃^ | 1464.57 | 1427.88 | cid-miR-23a-3-5p^＃^ | 1.64 | 1.46 |
| cid-miR-222b^＃^ | 1347.97 | 1453.23 | cid-miR-18b-3p^＃^ | 1.27 | 0.97 |
| cid-miR-30e-3p^＃^ | 1236.06 | 1155.29 | cid-miR-153c-5p^＃^ | 1.19 | 1.04 |
| cid-miR-126b-3p^＃^ | 1093.68 | 889.36 | cid-miR-10b-2-3p^＃^ | 1.19 | 0.83 |
| cid-miR-222a-3p^＃^ | 1062.09 | 725.71 | cid-miR-430a-3p^＃^ | 1.19 | 0.35 |
| cid-miR-16c-3p^＃^ | 805.64 | 754.18 | cid-miR-124-3p^＃^ | 0.89 | 0.56 |
| cid-miR-2188-3p^＃^ | 573.40 | 404.65 | cid-miR-363-5p^＃^ | 0.89 | 0.28 |
| cid-miR-15c^＃^ | 465.22 | 475.06 | cid-miR-30a-3p^＃^ | 0.82 | 0.69 |
| cid-miR-181a-2-3p^＃^ | 396.89 | 692.24 | cid-miR-727-5p^＃^ | 0.67 | 0.56 |
| cid-miR-29b^＃^ | 306.59 | 259.12 | cid-miR-137-3p^＃^ | 0.67 | 0.28 |
| cid-miR-27a-5p^＃^ | 198.63 | 221.42 | cid-miR-193a-5p^＃^ | 0.67 | 0.21 |
| cid-miR-210-5p^＃^ | 190.21 | 212.26 | cid-miR-132-5p^＃^ | 0.60 | 2.29 |
| cid-miR-26a-3p^＃^ | 178.52 | 183.86 | cid-miR-125c-3p^＃^ | 0.60 | 0.97 |
| cid-miR-139-5p^＃^ | 174.12 | 176.29 | cid-miR-1306^＃^ | 0.60 | 0.35 |
| cid-miR-27d^＃^ | 173.52 | 197.05 | cid-miR-193b-5p^＃^ | 0.52 | 0.28 |
| cid-miR-19c-3p^＃^ | 126.44 | 71.52 | cid-miR-430b-3p^＃^ | 0.45 | 0.56 |
| cid-miR-27b-5p^＃^ | 80.09 | 132.69 | cid-miR-31^＃^ | 0.37 | 0.83 |
| cid-miR-100-2-3p^＃^ | 76.29 | 72.97 | cid-miR-23a-5p^＃^ | 0.30 | 1.32 |
| cid-miR-18a^＃^ | 52.97 | 44.23 | cid-miR-139-3p^＃^ | 0.30 | 0.76 |
| cid-miR-125b-1-3p^＃^ | 52.23 | 73.81 | cid-miR-194b^＃^ | 0.30 | 0.49 |
| cid-miR-30c-3p^＃^ | 51.04 | 31.31 | cid-miR-96-3p^＃^ | 0.30 | 0.21 |
| cid-miR-9-4-3p^＃^ | 46.19 | 16.59 | cid-miR-10b-3p^＃^ | 0.30 | 0.07 |
| cid-miR-22b-5p^＃^ | 42.39 | 30.62 | cid-miR-9-3p^＃^ | 0.22 | 0.49 |
| cid-miR-22a-5p^＃^ | 38.37 | 38.95 | cid-miR-202-5p^＃^ | 0.22 | 0.42 |
| cid-miR-140-5p^＃^ | 37.92 | 27.29 | cid-miR-301c-5p^＃^ | 0.22 | 0.28 |
| cid-miR-20a-3p^＃^ | 37.18 | 19.65 | cid-miR-203b-5p^＃^ | 0.22 | 0.28 |
| cid-miR-141-3p^＃^ | 35.32 | 21.25 | cid-miR-200c-5p^＃^ | 0.15 | 0.21 |
| cid-miR-100-3p^＃^ | 35.09 | 70.06 | cid-miR-135a^＃^ | 0.15 | 0.14 |
| cid-miR-455-2-5p^＃^ | 31.96 | 32.36 | cid-miR-133c-3p^＃^ | 0.15 | 0.14 |
| cid-miR-221-5p^＃^ | 31.89 | 46.03 | cid-miR-9-7-3p^＃^ | 0.15 | 0.07 |
| cid-miR-107a-5p^＃^ | 31.44 | 31.66 | cid-miR-458-5p^＃^ | 0.15 | 0.07 |
| cid-miR-92a-5p^＃^ | 29.36 | 21.73 | cid-miR-153b-5p^＃^ | 0.15 | 0.07 |
| cid-miR-153b-3p^＃^ | 24.89 | 14.16 | cid-miR-1^＃^ | 0.15 | 0.07 |
| cid-miR-125b-3-3p^＃^ | 21.61 | 32.08 | cid-miR-15b-3p^＃^ | 0.07 | 0.35 |
| cid-miR-10c-3p^＃^ | 20.34 | 18.12 | cid-miR-200b-5p^＃^ | 0.07 | 0.14 |
| cid-miR-15a-3p^＃^ | 14.68 | 19.37 | cid-miR-206-3p^＃^ | 0.07 | 0.07 |
| cid-miR-128-5p^＃^ | 14.16 | 11.73 | cid-miR-203a-5p^＃^ | 0.07 | 0.07 |
| cid-miR-125b-2-3p^＃^ | 12.07 | 7.98 | cid-miR-129-1-3p^＃^ | 0.07 | 0.07 |
| cid-miR-26a-2-3p^＃^ | 11.92 | 13.68 | cid-miR-34c-5p^＃^ | 0.3 | 0 |
| cid-miR-181c-3p^＃^ | 10.51 | 7.57 | cid-miR-1788-3p^＃^ | 0.3 | 0 |
| cid-miR-19d-5p^＃^ | 9.98 | 5.55 | cid-miR-459-3p^＃^ | 0.15 | 0 |
| cid-miR-301b-3p^＃^ | 9.91 | 7.85 | cid-miR-430b-5p^＃^ | 0.15 | 0 |
| cid-miR-19a-5p^＃^ | 9.83 | 7.43 | cid-miR-733^＃^ | 0.07 | 0 |
| cid-miR-455-3p^＃^ | 9.76 | 12.5 | cid-miR-205-3p^＃^ | 0.07 | 0 |
| cid-miR-181b-3p^＃^ | 7.45 | 9.37 | cid-miR-138-3p^＃^ | 0.07 | 0 |
| cid-miR-135b-5p^＃^ | 7.08 | 7.15 | cid-miR-137-5p^＃^ | 0.07 | 0 |
| cid-miR-212^＃^ | 7.00 | 10.28 | cid-miR-10d-3p^＃^ | 0.07 | 0 |
| cid-miR-737-3p^＃^ | 6.78 | 2.78 | cid-miR-738^＃^ | 0 | 0.07 |
| cid-miR-17a-3p^＃^ | 6.63 | 4.10 | cid-miR-730^＃^ | 0 | 0.14 |
| cid-miR-460-5p^＃^ | 6.48 | 5.49 | cid-miR-729^＃^ | 0 | 0.07 |
| cid-miR-130c-5p^＃^ | 6.33 | 4.51 | cid-miR-728^＃^ | 0 | 0.07 |
| cid-let-7d-3p^＃^ | 6.18 | 7.98 | cid-miR-25-5p^＃^ | 0 | 0.21 |
| cid-let-7c-3p^＃^ | 6.18 | 7.98 | cid-miR-190b^＃^ | 0 | 0.07 |
| cid-miR-19b-5p^＃^ | 6.11 | 3.47 | cid-miR-182-3p^＃^ | 0 | 0.07 |
|  |  |  |  |  |  |
| cid-miR-143^※^ | 222118.58 | 288323.88 | cid-miR-29b3-3p^※^ | 336.65 | 277.47 |
| cid-miR-21^※^ | 86621.38 | 93020.24 | cid-miR-16a^※^ | 353.42 | 262.68 |
| cid-let-7a^※^ | 70576.41 | 64017.30 | cid-miR-456^※^ | 293.11 | 237.88 |
| cid-miR-25-3p^※^ | 63066.19 | 48310.44 | cid-miR-130b^※^ | 265.76 | 262.73 |
| cid-miR-26a-5p^※^ | 69249.00 | 51731.65 | cid-miR-458-3p^※^ | 259.88 | 338.90 |
| cid-miR-10c-5p^※^ | 54711.66 | 56931.00 | cid-miR-454b^※^ | 259.88 | 171.71 |
| cid-miR-462^※^ | 52392.88 | 65904.34 | cid-miR-27e^※^ | 255.48 | 200.66 |
| cid-miR-181a-5p^※^ | 44088.28 | 49097.67 | cid-miR-153c-3p^※^ | 248.18 | 165.32 |
| cid-miR-142a-5p^※^ | 32490.65 | 23792.57 | cid-miR-19d-3p^※^ | 243.56 | 215.59 |
| cid-miR-92a-3p^※^ | 29581.48 | 20523.47 | cid-miR-722^※^ | 239.91 | 235.03 |
| cid-miR-26b^※^ | 27128.89 | 16647.81 | cid-miR-23b^※^ | 238.94 | 292.45 |
| cid-miR-30d^※^ | 16688.71 | 16454.72 | cid-miR-204-5p^※^ | 233.95 | 133.94 |
| cid-miR-126a^※^-5p | 14291.10 | 10858.03 | cid-miR-217^※^ | 208.47 | 105.47 |
| cid-miR-101a^※^ | 12678.56 | 9155.74 | cid-miR-122^※^ | 178.00 | 72.07 |
| cid-miR-2188-5p^※^ | 12626.78 | 4765.93 | cid-miR-125c-5p^※^ | 173.52 | 178.93 |
| cid-miR-30e-5p^※^ | 12525.31 | 11275.32 | cid-miR-218a^※^ | 156.16 | 120.74 |
| cid-let-7g^※^ | 11432.37 | 11702.26 | cid-miR-737-5p^※^ | 151.69 | 139.56 |
| cid-miR-1388-5p^※^ | 9656.45 | 11916.46 | cid-miR-375^※^ | 148.57 | 42.28 |
| cid-miR-100-5p^※^ | 9619.79 | 9529.29 | cid-miR-93^※^ | 141.86 | 131.3 |
| cid-miR-146a^※^ | 8566.79 | 7039.78 | cid-miR-19a-3p^※^ | 140.37 | 97.34 |
| cid-miR-27b-3p^※^ | 7215.70 | 7981.71 | cid-miR-181c-5p^※^ | 130.16 | 101.51 |
| cid-let-7f^※^ | 6810.24 | 7809.72 | cid-miR-194a^※^ | 107.21 | 36.45 |
| cid-miR-192^※^ | 6197.20 | 1933.35 | cid-miR-24^※^ | 103.79 | 107.62 |
| cid-miR-142b-5p^※^ | 5974.8 | 5628.28 | cid-miR-214^※^ | 102.45 | 76.93 |
| cid-miR-22a-3p^※^ | 4991.76 | 5595.65 | cid-miR-18c^※^ | 100.21 | 39.99 |
| cid-miR-144-5p^※^ | 4724.66 | 1730.75 | cid-miR-187^※^ | 99.32 | 114.29 |
| cid-miR-30c-5p^※^ | 3962.83 | 3590.57 | cid-miR-182-5p^※^ | 83.45 | 31.18 |
| cid-miR-338^※^ | 3765.39 | 3529.47 | cid-miR-200a-3p^※^ | 72.20 | 52.35 |
| cid-miR-15b-5p^※^ | 3489.12 | 3298.82 | cid-miR-454a^※^ | 66.46 | 56.45 |
| cid-miR-146b^※^ | 3405.53 | 3258.06 | cid-miR-455-5p^※^ | 65.42 | 92.97 |
| cid-miR-27c-3p^※^ | 2810.60 | 2722.66 | cid-miR-20b-5p^※^ | 65.34 | 29.58 |
| cid-miR-16c-5p^※^ | 2599.30 | 2340.09 | cid-miR-429a^※^ | 54.91 | 44.30 |
| cid-miR-30b^※^ | 2550.87 | 2151.79 | cid-miR-301c-3p^※^ | 54.69 | 60.06 |
| cid-miR-210-3p^※^ | 2285.33 | 2440.98 | cid-miR-184^※^ | 48.95 | 62.77 |
| cid-miR-150^※^ | 2141.01 | 1068.92 | cid-miR-10d-5p^※^ | 48.80 | 34.44 |
| cid-miR-125a^※^ | 2092.06 | 1933.15 | cid-miR-190a^※^ | 47.53 | 29.02 |
| cid-miR-22b-3p^※^ | 2090.64 | 1627.02 | cid-miR-200b-3p^※^ | 30.10 | 25.76 |
| cid-let-7h^※^ | 2081.93 | 2201.85 | cid-miR-193b-3p^※^ | 29.95 | 15.69 |
| cid-miR-10b^※^-5p | 1678.33 | 1882.18 | cid-miR-92b-3p^※^ | 29.65 | 17.08 |
| cid-miR-145-5p^※^ | 1673.56 | 1843.02 | cid-miR-2187-5p^※^ | 27.34 | 33.26 |
| cid-miR-2184^※^ | 1623.27 | 1519.95 | cid-miR-138-5p^※^ | 26.97 | 10.21 |
| cid-let-7i^※^ | 1580.80 | 1016.29 | cid-miR-7132-5p^※^ | 26.18 | 28.33 |
| cid-let-7j^※^ | 1578.86 | 1750.40 | cid-miR-107b^※^ | 25.70 | 41.94 |
| cid-miR-125b-5p^※^ | 1563.21 | 1623.13 | cid-miR-460-3p^※^ | 25.03 | 29.58 |
| cid-miR-181b-5p^※^ | 1460.17 | 1425.31 | cid-miR-205-5p^※^ | 24.66 | 7.29 |
| cid-miR-221-3p^※^ | 1419.57 | 1286.80 | cid-miR-7147^※^ | 23.39 | 20.00 |
| cid-miR-199-3p^※^ | 1405.26 | 1324.36 | cid-miR-34a^※^ | 19.15 | 33.4 |
| cid-miR-199-5p^※^ | 1354.89 | 1237.71 | cid-miR-132-3p^※^ | 17.88 | 23.47 |
| cid-let-7d-5p^※^ | 1318.24 | 1124.6 | cid-miR-183-5p^※^ | 16.39 | 6.8 |
| cid-miR-99^※^ | 1277.11 | 1355.12 | cid-miR-218b^※^ | 16.24 | 13.4 |
| cid-miR-216b^※^ | 1173.77 | 693.91 | cid-miR-365^※^ | 15.12 | 13.61 |
| cid-miR-457a^※^ | 1135.10 | 1021.91 | cid-miR-200c-3p^※^ | 13.86 | 9.65 |
| cid-let-7b^※^ | 1104.70 | 983.65 | cid-miR-9-5p^※^ | 12.59 | 6.67 |
| cid-miR-148^※^ | 1078.03 | 930.61 | cid-miR-212-5p^※^ | 12.19 | 23.64 |
| cid-miR-29a^※^ | 1040.11 | 994.14 | cid-miR-18b-5p^※^ | 12.00 | 10.28 |
| cid-miR-128-3p^※^ | 1036.08 | 1060.38 | cid-miR-724^※^ | 11.92 | 15.41 |
| cid-let-7c-5p^※^ | 1027.07 | 1044.89 | cid-miR-193a-3p^※^ | 11.85 | 8.33 |
| cid-miR-222a-5p^※^ | 979.91 | 1533.28 | cid-miR-725-3p^※^ | 9.24 | 0.97 |
| cid-miR-27a-3p^※^ | 852.20 | 885.27 | cid-miR-734^※^ | 7.38 | 10.9 |
| cid-miR-10a-5p^※^ | 831.86 | 1071.69 | cid-miR-7133-3p^※^ | 6.71 | 3.27 |
| cid-miR-15a-5p^※^ | 732.40 | 748.62 | cid-miR-203b-3p^※^ | 6.63 | 0.97 |
| cid-miR-103^※^ | 660.65 | 545.88 | cid-miR-133a-3p^※^ | 6.33 | 9.23 |
| cid-miR-140-3p^※^ | 649.92 | 468.67 | cid-miR-499-5p^※^ | 6.03 | 4.58 |
| cid-miR-301a^※^ | 635.98 | 605.45 | cid-miR-203a-3p^※^ | 5.66 | 0.83 |
| cid-miR-17a-5p^※^ | 628.16 | 419.72 | cid-miR-96-5p^※^ | 3.43 | 0.42 |
| cid-miR-19b-3p^※^ | 616.69 | 454.71 | cid-miR-7a^※^ | 3.20 | 1.74 |
| cid-miR-152^※^ | 546.80 | 521.44 | cid-miR-726^※^ | 2.53 | 1.87 |
| cid-miR-23a-3p^※^ | 531.30 | 557.06 | cid-miR-129-5p^※^ | 2.46 | 1.46 |
| cid-miR-457b-5p^※^ | 483.84 | 410.21 | cid-miR-196a-5p^※^ | 2.31 | 1.11 |
| cid-miR-101b^※^ | 473.86 | 303.21 | cid-miR-153a-3p^※^ | 2.16 | 0.35 |
| cid-miR-30a-5p^※^ | 460.45 | 450.55 | cid-miR-429b^※^ | 1.94 | 1.6 |
| cid-miR-216a^※^ | 452.70 | 209.55 | cid-miR-135c^※^ | 1.79 | 0.97 |
| cid-miR-107a-3p^※^ | 441.97 | 482.14 | cid-miR-489^※^ | 1.34 | 1.32 |
| cid-miR-363-3p^※^ | 420.96 | 159.14 | cid-miR-727-3p^※^ | 1.27 | 1.11 |
| cid-miR-130a^※^ | 420.51 | 355.22 | cid-miR-459-5p^※^ | 1.27 | 0.62 |
| cid-miR-20a-5p^※^ | 401.22 | 292.45 | cid-miR-196b^※^ | 1.27 | 2.22 |
| cid-miR-155^※^ | 382.07 | 449.16 | cid-miR-153b^※^ | 27.00 | 15.17 |
| cid-miR-130c-3p^※^ | 355.47 | 301.34 | cid-miR-133b-3p^※^ | 0.15 | 0.28 |
| cid-miR-457b-3p^※^ | 354.57 | 325.57 |  |  |  |
|  |  |  |  |  |  |
| cid-miR-7552a-5p^△^ | 4.34 | 2.16 | cid-miR-764^△^ | 20.45 | 13.31 |
| cid-miR-29c^△^ | 89.91 | 70.78 | cid-miR-677-5p^△^ | 0.57 | 0.82 |
| cid-miR-551-3p^△^ | 23.4 | 8.77 | cid-miR-626^△^ | 0.08 | 1.78 |
| cid-miR-455b^△^ | 35.10 | 34.65 | cid-miR-6165^△^ | 7.94 | 6.32 |
| cid-miR-6157^△^ | 0.74 | 1.71 | cid-miR-5674b^△^ | 1.80 | 1.56 |
| cid-miR-3120^△^ | 2.29 | 1.64 | cid-miR-4956-3p^△^ | 2.45 | 1.26 |
| cid-miR-31-3p^△^ | 0.98 | 0.74 | cid-miR-36b-5p^△^ | 2.05 | 0.89 |
| cid-miR-132b^△^ | 2.54 | 2.45 | cid-miR-34a-3p^△^ | 6.87 | 7.58 |
| cid-miR-132-2-5p^△^ | 21.11 | 38.96 | cid-miR-3428^△^ | 4.01 | 6.47 |
| cid-miR-3618^△^ | 21.6 | 18.81 | cid-miR-30a-2-5p^△^ | 4.83 | 3.57 |
| cid-miR-9771^△^ | 2.95 | 2.90 | cid-miR-29b-2^△^ | 0.33 | 0.67 |
| cid-miR-96-2-3p^△^ | 7.20 | 2.53 | cid-miR-1829a-3p^△^ | 2.37 | 1.71 |
| cid-miR-9292^△^ | 1.72 | 0.52 | cid-miR-153-5p^△^ | 7.61 | 2.53 |
| cid-miR-9005^△^ | 0.16 | 0.97 | cid-miR-151^△^ | 0.74 | 1.04 |
| cid-miR-866-5p^△^ | 1.15 | 0.22 | cid-miR-1392-5p^△^ | 2.05 | 1.34 |
| cid-miR-8435-3p^△^ | 1.80 | 3.27 | cid-miR-1097^△^ | 1.23 | 0.59 |
| cid-miR-8266-5p^△^ | 0.57 | 0.45 | cid-let-7c-2-5p^△^ | 0.82 | 0.59 |
| cid-miR-8163-5p^△^ | 1.88 | 1.64 |  |  |  |

**Note:** # miRNAs that match perfectly known zebrafish miRNAs in miRBase (22.1).

※miRNAs that match known zebrafish miRNAs in miRBase (22.1) but are mutations at the end of mature sequences or differences in genomic localization of precursor sequences.

△miRNAs that are homologous with known miRNA in other animals except zebrafish in miRBase (22.1).
