# Supplementary material for: Expression characteristics and interaction networks of microRNAs in spleen tissues of grass carp (Ctenopharyngodon idella)
Source: PLoS One. 2022 Mar 28;17(3):e0266189. doi: 10.1371/journal.pone.0266189 (PMC8959171; doi:10.1371/journal.pone.0266189)
Supplement: S2 Table — (DOCX) [file pone.0266189.s004.docx]

**S2 Table. List of novel miRNAs identified in grass carp spleen.**

| **miRNA name** | **TPM value(cis1)** | **TPM value(cis3)** | **Mature sequence** | **Precursor sequence** | **Precursor coordinate** |
| --- | --- | --- | --- | --- | --- |
| cid-miR-N1 | 62.34 | 12.49 | uuaauugggacacuuuuugcc | uuaauugggacacuuuuugccauugugaugaccaaagaaaagucccaauuggcc | 10:38580205..38580259:+ |
| cid-miR-N2 | 4.50 | 2.16 | cccguguggcuuaugagaucc | gucuuaucagccguaccuugcugcugcuugacagcccguguggcuuaugagaucc | 8:19139731..19139786:- |
| cid-miR-N3 | 4.17 | 9.29 | aaucccagcuguguguuugugc | aaucccagcuguguguuugugcaucagaacauuucagacgucacagcuggaga | 3:4220080..4220133:+; |
|  |  |  |  | aaucccagcuguguguuugugcaucagaacauuucagacaucacagcuggagaga | 3:3861231..3861286:+ |
| cid-miR-N4 | 2.37 | 2.97 | aaccaaucagagcucagagca | aaccaaucagagcucagagcaccagcugaccucgucacuaaaggagcucgaguucuggugaguaac | 12:48233258..48233324:- |
| cid-miR-N5 | 2.13 | 1.19 | auaccagguccuguaagcuu | gcagggucgggccugguuaguacuuggaugggagaccgccugggaauaccagguccuguaagcuu | 4:39704927..39704992:- |
| cid-miR-N6 | 2.13 | 3.57 | cuauggaagucaauggggucc | acacauugacuuccauuuuuuccuucuauggaagucaauggggucc | 19:22728800..22728846:- |
| cid-miR-N7 | 1.31 | 0.52 | uuuucucauugcacugcagu | uuuucucauugcacugcagugaaacaucugaucuucauuguccuguuacuuacugcagcacaaucagaaaauu | 16:51761468..51761541:+ |
| cid-miR-N8 | 1.15 | 1.19 | uguacuaccaguaguuuucugca | uguacuaccaguaguuuucugcaccuuuucaucuuuuaagaaaacucuggcagcacaga | 18:30023585..30023644:- |
| cid-miR-N9 | 0.49 | 0.52 | uccaucagucacgugaccuacc | gaggucacgcgagugguggcaggguuaccacaaacuaaccgaaucuuccaucagucacgugaccuacc | 5:35345794..35345862:- |
