# Supplementary material for: Expression characteristics and interaction networks of microRNAs in spleen tissues of grass carp (Ctenopharyngodon idella)
Source: PLoS One. 2022 Mar 28;17(3):e0266189. doi: 10.1371/journal.pone.0266189 (PMC8959171; doi:10.1371/journal.pone.0266189)
Supplement: S3 Table — (DOCX) [file pone.0266189.s005.docx]

**S3 Table. The significant differentially expressed miRNAs identified in this study.**

| **miRNA_name** | **cis1_TPM value** | **cis3_TPM value** | **log2(Fold_change)** | **p-value** | **q-value** |
| --- | --- | --- | --- | --- | --- |
| cid-miR-192 | 6197.20 | 1933.35 | -1.673197057 | 0 | 0 |
| cid-miR-144-5p | 4724.66 | 1730.75 | -1.441496529 | 0 | 0 |
| cid-miR-2188-5p | 12626.78 | 4765.93 | -1.398340477 | 0 | 0 |
| cid-miR-451 | 36096.07 | 16792.92 | -1.096672023 | 0 | 0 |
| cid-miR-144-3p | 3317.98 | 1091.20 | -1.597073005 | 5.28E-256 | 9.84E-255 |
| cid-miR-363-3p | 420.96 | 159.14 | -1.396070014 | 3.43E-28 | 2.60E-27 |
| cid-miR-216a | 452.70 | 209.55 | -1.10394415 | 2.15E-21 | 1.52E-20 |
| cid-miR-375 | 148.57 | 42.28 | -1.805778912 | 3.28E-15 | 2.03E-14 |
| cid-miR-122 | 178.00 | 72.07 | -1.297089855 | 1.32E-11 | 6.77E-11 |
| cid-miR-194a | 107.21 | 36.45 | -1.549132124 | 1.93E-09 | 8.80E-09 |
| cid-miR-18c | 100.21 | 39.99 | -1.317998661 | 2.75E-07 | 1.08E-06 |
| cid-miR-182-5p | 83.45 | 31.18 | -1.412974486 | 7.56E-07 | 2.92E-06 |
| cid-miR-9-4-3p | 46.19 | 16.59 | -1.469950025 | 0.00015023 | 0.00048864 |
| cid-miR-20b-5p | 65.34 | 29.58 | -1.136027767 | 0.000222556 | 0.000712581 |
| cid-miR-205-5p | 24.66 | 7.29 | -1.750865445 | 0.001676291 | 0.00464186 |
| cid-miR-138-5p | 26.97 | 10.21 | -1.39405602 | 0.005379672 | 0.014133038 |
| cid-miR-551-3p | 23.40 | 8.77 | -1.369374124 | 0.011069985 | 0.014218901 |
| cid-miR-725-3p | 9.24 | 0.97 | -3.244519564 | 0.005931888 | 0.01519418 |
| cid-miR-212-5p | 12.19 | 23.64 | 1.002017567 | 0.043009481 | 0.04265143 |
| cid-miR-100-3p | 35.09 | 70.06 | 1.004847676 | 0.00052425 | 0.001579806 |
