# Supplementary material for: Expression characteristics and interaction networks of microRNAs in spleen tissues of grass carp (Ctenopharyngodon idella)
Source: PLoS One. 2022 Mar 28;17(3):e0266189. doi: 10.1371/journal.pone.0266189 (PMC8959171; doi:10.1371/journal.pone.0266189)
Supplement: Attachment — Submitted filename: Response to Reviewers.docx [file pone.0266189.s006.docx]

**Response to** **Review Comments**

Dear editor and reviewers,

Thank you very much for your hard work on my manuscript. According to the review comments of editors and reviewers and the requirements of the magazine, we have made a thorough revision of the manuscript entitled “Expression characteristics and interaction networks of microRNAs in spleen tissues of grass carp (*Ctenopharyngodon idella*)” (PONE-D-21-39912). **The sections marked in red are the modified contents in the “Revised Manuscript with Track Changes”**.

We hope that our revised manuscript could be accepted for publication.

Sincerely,

Guoxi Li

**The response to Journal Requirements**

**Point 1:** Please ensure that your manuscript meets PLOS ONE's style requirements, including those for file naming.

**Response 1:** We have revised our manuscript completely according to PLOS ONE's style requirements, including figures, table, references, language,and etc.. See the revised text.

**Point 2:** We suggest you thoroughly copyedit your manuscript for language usage, spelling, and grammar.

**Response 2:** Our original manuscript has been sent to Asia Science Editing for the English language editing before the initial submission. The language of revised manuscript has been refined and polished by Dr. Xiaojun Liu ( Henan Agricultural University). Therefore, we believe that the language of our manuscript can meet the requirements of the magazine.

**Point 3:** We note that the grant information you provided in the ‘Funding Information’ and ‘Financial Disclosure’ sections do not match.

**Response 3:** I'm sorry, this is caused by the operation error. In fact, we have provided the correct grant numbers in the ‘Funding Information’ section. But we mismanaged in the ‘Financial Disclosure’ sections. **We have amended "Funding Statement" within our cover letter as follows**:

"**This work was supported by grants from the Project of Henan University of Technology（CN）(No.2017XTCX04).**"

Please help to change it online. Thank you!

**Point 4:** We note that you have provided funding information that is not currently declared in your Funding Statement. However, funding information appears in the Acknowledgments section.

**Response 4:** According to your comments, we have removed any funding-related text from the manuscript. We would like to update our Funding Statement as follows:

"**This work was supported by grants from the Project of Henan University of Technology（CN）(No.2017XTCX04).**"

Please help to change it online. Thank you!

**Point 5:** All data and its representation including raw sequencing data (NCBI) should be accessible for the reviewers.

**Response 5:** We have declared that all data are fully available without restriction in Data Availability. At the same time, we also state the following in the Data Availability Statement section of our manuscript:

"The raw sequencing data had been deposited into the NCBI database Sequence Read Archive under number PRJNA328412 (mRNA) and PRJNA645243 (miRNA). The other data and material used and analyzed in this study are within the manuscript and its Supporting Information files."

Therefore, all data for this study are available.

However, the Reviewer#2 mentioned that no entry was found using accession number PRJNA645243, and the main reason was the data release date setting problem. The data was originally set to be released on July 7, 2024. To this end, we have made corresponding adjustments.

See: https://www.ncbi.nlm.nih.gov/bioproject/PRJNA645243

**The response to comments from Reviewer #1**

In their manuscript , Yinli et al. studied miRNA transcriptome and miRNA–mRNA interaction networks in grass carp normal spleen tissue. This study is descriptive and provides resources for understanding the molecular details of the spleen development in the grass carp. The presented conclusions are well supported by the data. Appropriate controls have been used to support the experiments.

**Response:** Thank you very much for your objective evaluation of our manuscript.

**The response to comments from Reviewer #2**

Thank you for your objective evaluation of our manuscript. We have revised the manuscript in accordance with your comments.

**Point 1:** Please rectify the raw data archive accession number. The information is submitted which says, “The raw sequencing data had been deposited into the NCBI database Sequence Read Archive under number PRJNA328412 (mRNA) and PRJNA645243 (miRNA)”. However, no entry was found using accession number PRJNA645243. Inaccessibility of the data may impede the conclusions of the paper.

**Response 1:** I'm sorry. The main reason was the data release date setting problem. The data was originally set to be released on July 7, 2024. To this end, we have made corresponding adjustments.

See: https://www.ncbi.nlm.nih.gov/bioproject/PRJNA645243

**Point 2:** Figures are illegible, specifically Figures 1,3,5 and 6 are inaccessible to make any assessment of the data presented. Other figures also need significant improvement in presentations.

**Response 2:** I am very sorry for the trouble caused to your [view](javascript:;)ing. This may be caused by file compression when the system generates PDF. In fact, the original image downloaded from the submission system is quite clear.

According to your comments, we have improved all the figures, further increased the resolution and changed the presentations of some figures.

[In](javascript:;) [addition](javascript:;), the figure 1and figure 3 in the original version of the manuscript have been used as supporting information to simplify and refine our manuscript.

**Point 3:** Figure 4: Please provide the qPCR primer sequences or catalog number if commercially available.

**Response 3:** The qRT-PCR analysis for validating six SDE miRNAs was performed using the kits customized at the RiboBio company (Guangzhou, China). The qPCR primer sequences were designed by the company based on the mature sequences and precursor sequence of the identified miRNAs. However, due to intellectual property rights, the company did not provide us with primers related information. Therefore, we are very sorry that we cannot provide these primer sequences in manuscript.

**Point 4:** Line 17 and 99: Six versus two library construction were respectively mentioned. Authors needs to clarify the differences.

**Response 4:** I'm very sorry, this is due to our spelling mistake. In fact, we constructed two libraries in this study. We have revised this error, see the line 19 in the revised text.

**The response to comments from Reviewer #3**

Thank you very much for your objective evaluation of our manuscript. We have revised the manuscript in accordance with your comments.

**Point 1:** The main conclusion from the abstract and the introduction stresses upon the novel finding of the miRNA transcriptome and miRNA-mRNA interaction network, whereas in the discussion section the conclusion was stressed upon miRNA regulation of the lipid metabolism and spleen function. Kindly rearrange such that a common conclusion is presented throughout the manuscript.

**Response 1:** Thank you for your comments. According to your opinion, we have deleted the content relating to lipid metabolism in the line 517-527 of paragraph 5, line 541-546 of paragraph 6, and the last paragraph in the discussion section of original manuscript. Thus, the main conclusion throughout the manuscript stresses upon the characteristics of the miRNA transcriptome profile and miRNA-mRNA interaction network in the spleen. At the same time, we also adjusted the corresponding references. See the revised text.

**Point 2:** In Line 99, it says “two miRNA libraries were constructed from the spleen tissue of one year old and three year old”, whereas in line 140 it says “ six miRNA libraries were constructed from spleen tissue of grass carp at one year and three years of age”. So what is the exact number of libraries being constructed?

**Response 2:** I'm very sorry, this is due to our spelling mistake. In fact, we constructed two libraries in this study. We have revised this error, see the line 168 in the revised text.

**Point 3:** The resolution of the figures need further enhancement, as it is difficult to visualize and read.

**Response 3:** I am very sorry for the trouble caused to your [view](javascript:;)ing and reading. This may be caused by file compression when the system generates PDF. In fact, the original image downloaded from the submission system is quite clear.

According to your comments, we have improved all the figures, further increased the resolution and changed the presentations of some figures.

[In](javascript:;) [addition](javascript:;), the figure 1and figure 3 in the original version of the manuscript have been used as supporting information to simplify and refine our manuscript.

**Point 4:** Although, it is mentioned that RNA isolation from spleen tissues was carried out using Trizol. But what type of method was used to homogenize the spleen tissues?

**Response 4:** The spleen tissues were homogenized by grinding in a mortar. We have supplemented this method in the "Sample collection and RNA isolation" [section](javascript:;), see the line 158-159 in the revised text.

**Point 5:** What is the amount of total RNA used to make each miRNA library construction by TruSeq Small RNA Sample prep Kit for sequencing purposes?

**Response 5:** The initial total RNA was 1μg for each miRNA library construction. We have supplemented this description in the "MiRNA library construction and post-sequencing analysis" [section](javascript:;), see the line 171 in the revised text.

**Point 6:** For qRT-PCR method section, kindly provide a brief description on how the cDNA was made? Also please include a reference of the method used for calculation of the miRNA expression level.

**Response 6:** Thank you for your comments. According to your comments, we have supplemented a brief description of cDNA synthesis in the "Quantitative real-time PCR (qRT-PCR) analysis" [section](javascript:;), see the line 249-253 in the revised text. At the same time, we also added a reference of the method used for calculation of the miRNA expression level, see the line 260 and line 786-788 in the revised text.

**Point 7:** Kindly maintain a uniformity of the numbering for figures including legends and text in the results section. For instance, Figure 1A is presented differently in the figure legends as (a) instead of (A), and the same is for figure 3, 4, and 6.

**Response 7:** We have revised them according to your comments. See the revised text.

**Point 8:** The subfigure number A, B, C,… and F should be also mentioned in the legend for figure 4 along with the respective miRNA.

**Response 8:** We have revised them according to your comments. See the line 363-366 in the revised text.

**Point 9:** The figure legends for figures 7, 8 and 9, should come after the result description, but not before as presented in the manuscript.

**Response 9:** Thank you so much for your comments. We have adjusted them according to your opinion. See the line 407-469 in the revised text.

**Point 10:** Lines 380, 391 and 400, the sentences start with the same words “ In the miRNA-mRNA interaction networks associated with”, kindly reframe each of the sentences differently.

**Response 10:** Thank you so much for your comments. We have revised these sentences according to your opinion. See the line 411, line 429 and line 452 in the revised text.
